# Serology- and Blood-PCR-Based Screening for Schistosomiasis in Pregnant Women in Madagascar—A Cross-Sectional Study and Test Comparison Approach

**DOI:** 10.3390/pathogens10060722

**Published:** 2021-06-08

**Authors:** Tanja Hoffmann, Imke Carsjens, Raphaël Rakotozandrindrainy, Mirko Girmann, Njary Randriamampionona, Oumou Maïga-Ascofaré, Andreas Podbielski, Andreas Hahn, Hagen Frickmann, Norbert Georg Schwarz

**Affiliations:** 1 Department of Microbiology and Hospital Hygiene, Bundeswehr Hospital Hamburg, 20359 Hamburg, Germany; tanja1hoffmann@bundeswehr.org (T.H.); imke.carsjens@gmx.de (I.C.); frickmann@bnitm.de (H.F.); 2 Department of Microbiology and Parasitology, University of Antananarivo, BP 566 Antananarivo, Madagascar; rakrapha13@gmail.com (R.R.); njrandriamampionona@gmail.com (N.R.); 3 Independent Researcher, 20095 Hamburg, Germany; microthewave@hotmail.com (M.G.); schwarznorbert@web.de (N.G.S.); 4 Infectious Disease Epidemiology Department, Bernhard Nocht Institute for Tropical Medicine Hamburg, 20359 Hamburg, Germany; maiga@bnitm.de; 5 Institute for Medical Microbiology, Virology and Infectious Disease Epidemiology, University Medicine Rostock, 18057 Rostock, Germany; hahn.andreas@me.com

**Keywords:** schistosomiasis, blood samples, Madagascar, pregnancy, test comparison, epidemiology

## Abstract

This work was conducted as a cross sectional study to define the disease burden of schistosomiasis in pregnant Madagascan women and to evaluate serological and molecular diagnostic assays. A total of 1154 residual EDTA blood samples from pregnant Madagascan women were assessed. The nucleic acid extractions were subjected to in-house real-time PCRs specifically targeting *S. mansoni* complex, *S. haematobium* complex, and African *Schistosoma* spp. on genus level, while the EDTA plasma samples were analyzed using *Schistosoma*-specific IgG and IgM commercial ELISA and immunofluorescence assays. The analyses indicated an overall prevalence of schistosomiasis in Madagascan pregnant women of 40.4%, with only minor regional differences and differences between serology- and blood PCR-based surveillance. The *S. mansoni* specific real-time PCR showed superior sensitivity of 74% (specificity 80%) compared with the genus-specific real-time PCR (sensitivity 13%, specificity 100%) in blood. The laborious immunofluorescence (sensitivity IgM 49%, IgG 87%, specificity IgM 85%, IgG 96%) scored only slightly better than the automatable ELISA (sensitivity IgM 38%, IgG 88%, specificity IgM 78%, IgG 91%). Infections with *S. mansoni* were detected only. The high prevalence of schistosomiasis recorded here among pregnant women in Madagascar calls for actions in order to reduce the disease burden.

## 1. Introduction

Schistosomiasis is highly prevalent in Madagascar [1]. Especially in remote areas, signs of periportal fibrosis associated with *Schistosoma (S.) mansoni* infections, even in school children, indicate high infection pressure and pathogen load [2]. This is particularly important considering the fact that liver alterations are usually observed as long-term consequences of *S. mansoni* infections [3]. Considerable infection rates in Madagascan children have been confirmed by numerous epidemiological assessments [4,5]. Abundance of well-adapted freshwater snails as intermediate hosts [6,7,8], associated with measurable pathogen DNA levels in freshwater [9], support the transmission of *Schistosoma* spp.

In Madagascar, *S. haematobium*, which is predominant in the Northern and Western regions of the island, is the major cause of urogenital schistosomiasis [10,11,12,13,14], potentially resulting in complications during pregnancy [15]. *S. haematobium* is generally assumed to be a relevant cause of obstetrical complications like ectopic pregnancy [16], preterm delivery, and reduced birth weight [17,18,19]. Indirect complications like altered immune responses or consequences of maternal anemia during pregnancy are discussed for newborns from mothers with *S. mansoni* infections as well [18,20,21,22,23,24].

So far, little is known about the epidemiology of schistosomiasis in Madagascan pregnant women. Between April and July 2010, a total of 1244 EDTA blood samples were collected and aliquots of each sample were subjected to urea pretreatment in order to stabilize nucleic acids within the samples. Afterwards, the samples were subjected to serological and PCR-assessment targeting a variety of pathogens, including plasmodiae, rickettsiae, *Treponema pallidum*, human immunodeficiency virus, cyclovirus, Zika virus, Dengue virus, Chikungunya virus, and Rift valley fever virus [25,26,27,28,29,30].

Next to microscopy and PCR from stool [4] as well as microscopy, PCR, and antigen testing from urine [31,32], schistosomiasis can also be detected by highly sensitive real-time PCR assays [33,34,35] from blood and previous infections by serology [36]. While serology is poorly suited to differentiate active from successfully cured schistosomiasis [36], recent assessments indicated steadily increasing cycle threshold values (Ct) of real-time PCR in the course after successful treatment [37], making PCR potentially suitable as a marker for disease activity.

In this study, the above-mentioned residual materials from blood samples from Madagascan pregnant women were assessed for active or previous schistosomiasis by serology and PCR. The aims were both a general epidemiologic assessment and a comparative assessment of the applied diagnostic approaches. Thereby, determination of potential cross-reactivity between anti-malaria-antibodies and anti-*Schistosoma*-antibodies [38], which was suggested from previous assessments [39,40,41], were considered. Finally, the diagnostic accuracy adjusted overall prevalence [42] was calculated considering the observed sensitivities and specificities of the test approaches.

## 2. Results

### 2.1. Local Epidemiology

**Figure 1:**
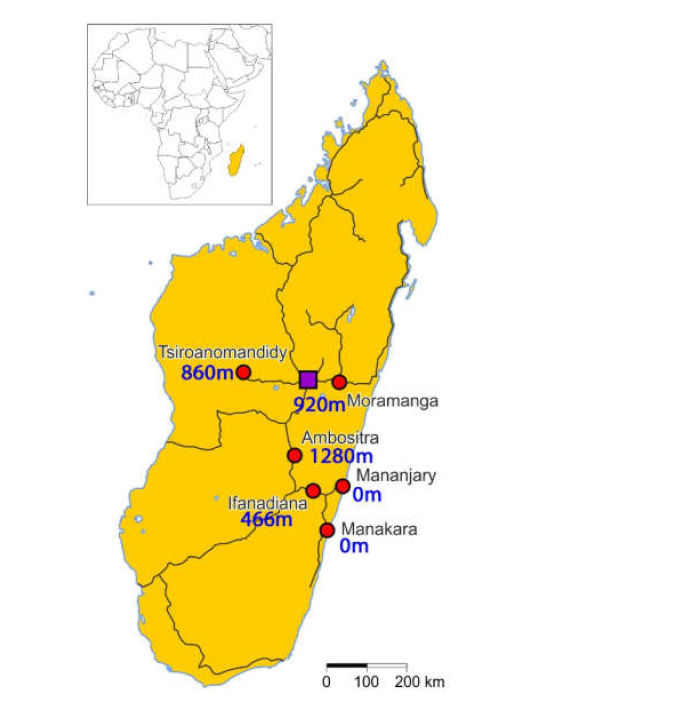
Map of Madagascar showing the 6 study locations and their altitude. The purple square is the capital Antananarivo; the dark lines represent main roads. The study found no signs of *S. haematobium* but demonstrated a presence of *S. mansoni* in all 6 locations [29].

After exclusion of 90 pregnant women (please see Appendix A for the geographic distribution), for whom the whole residual sample material was gone, the study population consisted of 1154 pregnant women from 6 different locations in Madagascar, 2 coastal, and 4 in the highlands (Figure 1). The median age was 25 in a right-skewed distribution and did not relevantly differ for the various study sites as detailed elsewhere [25,26,27,28,29,30]. Data on previous treatment for schistosomiasis were not available.

Samples from these 1154 pregnant women were investigated for *Schistosoma* spp.-specific DNA using real-time PCR and for IgM and IgG antibodies against schistosomiasis using serological methods (ELISA and immunofluorescence). Three different real-time PCR assays were compared, one species-overarching assay targeting the ITS-2 region for African *Schistosoma* spp. in general, one species-specific assay targeting the highly repetitive *Dra1* region for *S. haematobium* complex, and a species-specific assay targeting the highly repetitive *Sm1-7* region for *S. mansoni* complex. The latter two assays were run in a one-tube duplex approach. None of the *S. haematobium* PCRs were positive. The non-species-specific *Schistosoma* spp. PCR was positive in 5.5% of the samples, while the more sensitive *S. mansoni* specific PCR was positive in 42.0% of the samples.

The highest proportions of positive PCR samples (69.1%) were found in Ambositra, the highest study location (1280 m) (Table 1). Over all 6 sites, IgG seroprevalences were 37.3% with the immunofluorescence assay and 40.8% with the ELISA, while IgM seroprevalences were 28.5% with the immunofluorescence assay and 28.8% with the ELISA, respectively. Consistent with PCR results, the highest IgG-seroprevalence (71.1%) was found in Ambositra. Only a weak to moderate association between altitude and schistosomiasis prevalence could be demonstrated for the assessed population in correlation analysis, while a clear-cut correlation could not be shown (Table 1).

### 2.2. Descriptive Comparison of the Test Results Applying Different Assays

When comparing the test results of the serological methods (immunofluorescence vs. ELISA), 15.7% and 17.8% of the test comparisons based on positive or negative results (excluding borderline) according to the manufacturer’s instructions were discordant (one test negative and the other positive). Cohens-Kappa values (Landis and Koch, 1977 [43]) were between 55% and 70% (Table 2).

Although serological examinations and PCR are entirely different diagnostic methods, at least outside high-endemic areas, one may want to compare IgM-serology and PCR as both methods may be seen as indicators for an acute schistosomiasis infection requiring praziquantel treatment. We therefore also compared serological methods looking for IgMs with PCRs. As none of the *S. haematobium*-specific PCRs emerged positive, it was excluded from this analysis. The overall poor inter-rater reliability measures between the different methods that could be seen as indicators for acute schistosomiasis infections is quite sobering (Table 3).

**Figure 2:**
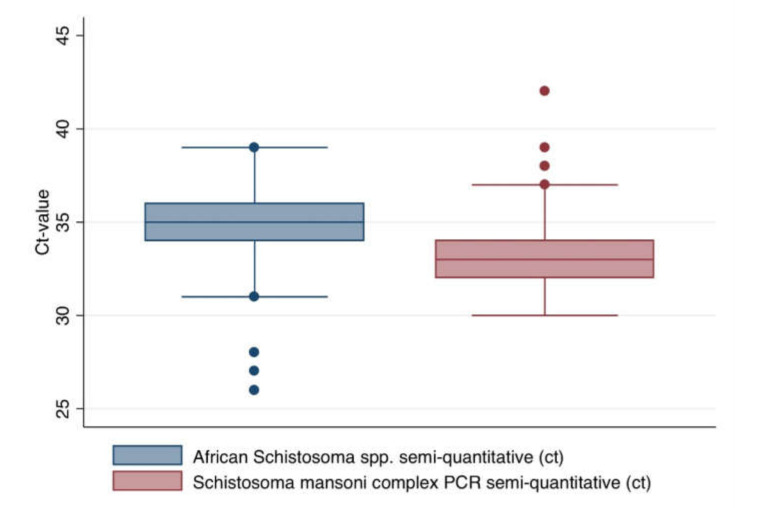
Comparison of cycle threshold values for the species-overarching PCR for *Schistosoma* spp. and the *S. mansoni*-specific PCR for all concordantly positive results.

For the concordantly positive PCRs, we compared the Ct-values (Figure 2). As expected, a tendency for lower Ct values was seen with the more sensitive *S. mansoni* complex-specific PCR approach, but with widely overlapping confidence intervals.

### 2.3. Latent Class Assessment Based Calculation of the Test Characteristics and the Resulting Diagnostic-Accuracy Adjusted Overall Prevalence

Applying latent class analysis on serological and PCR assays, both serological approaches for anti-*Schistosoma*-IgM scored with sensitivity < 50% and specificity < 90%. Thereby, immunofluorescence was slightly more reliable than the ELISA approach. Focusing on IgG-antibodies, sensitivity of both approaches was in a similar range > 85%, with specificity of ELISA > 90% and of immunofluorescence even >95%. Focusing on PCR, ITS-2-based genus specific PCR suggested low sensitivity < 15% but close-to perfect specificity. In comparison, *S. mansoni* complex-specific PCR with the multi-copy target *Sm1-7* showed considerably better sensitivity of 74%, but with reduced specificity close to 80%. Considering those test-specific features, a diagnostic accuracy adjusted overall prevalence of 40.4% in the assessed population was calculated. Details are provided in Table 4.

### 2.4. Estimation of Potential Influence of Cross-Reacting Anti-Malarial Antibodies

Making effects of cross-reacting anti-malarial antibodies unlikely, the phi coefficient for the serological assessments for antimalarial antibodies without further differentiation of antibody-subpopulations as previously described [29] and for the presently assessed anti-*Schistosoma*-antibodies scored in a similar range as the phi coefficient for malaria PCR [29] and for schistosomiasis PCR. Especially for the PCR approaches, cross-reactivity is unlikely for methodological reasons. Due to the weak association between altitude and schistosomiasis prevalence, individual phi-values for different study sites were not calculated. Details are provided in Table 5.

## 3. Discussion

The primary objective of the study was the assessment of the prevalence of previous or active schistosomiasis by serology [36], as well as likely active infection [37] by real-time PCR in Madagascan pregnant women. Of note, blood PCR for schistosomiasis can stay positive for a limited period of time after successful therapy as well [37]. The diagnostic accuracy adjusted prevalence estimation [44] suggested an overall prevalence of 40.4%. This high percentage is, interestingly, even lower than in previous reports from other assessments in Madagascar targeting other subpopulations [1,2,4,5]. The reasons for the surprisingly low IgG prevalence compared to the PCR results remain unresolved, although a considerable proportion of new infections, in which seroconversion had not occurred yet, cannot be completely excluded. Another likely reason is the previously described phenomenon of reduced antibody responses against *Schistosoma* spp. in pregnancy as described in 2009 [45]. Of note, only moderately differing percentages were recorded at different assessed study sites and both serology and PCR pointed in a similar direction regarding the proportion of positive samples. Focusing on the altitude of the different study sites, no strong correlations have been seen in spite of P-values suggesting significance for a weak association. At one side at sea level, interestingly, seropositivity rate was particularly low with relatively high IgM prevalence compared to IgG as well as comparably high rates of positive PCR. This composition of results could indicate active transmission of infections. As suggested by species complex-specific PCR, *S. mansoni* was the only detectable pathogen, while serological assessment did not allow discrimination on species level. The lacking detection of *S. haematobium*-specific DNA is in line with previous reports [10,11,12,13,14], suggesting endemicity of *S. haematobium* in the Northern and Western regions of Madagascar. Those regions were not in the focus of the presented study.

Although praziquantel therapy of schistosomiasis during pregnancy is recommended by the World Health Organization (WHO) [46], nationwide screening and screening-based therapy for pregnant women is so far not established in resource-poor Madagascar. At present, investigations on the suitability of test-and-treat strategies for the reduction of the burden of schistosomiasis in pregnant Madagascan women are underway [47].

Generally, species-specific PCR for highly repetitive multi-copy targets like *Sm1-7* [33] or *Dra1* [34] is considered more sensitive than genus-specific PCR targeting less repetitive genetic elements like ITS-2 [31] and is thus usually applied for PCR from blood [33,35]. When comparing Ct-values obtained by species-specific and genus-specific PCRs, widely overlapping confidence intervals counteracted a tendency for the expected lower Ct-values in case of species complex-specific PCR with specificity for a multi-copy target. Further, latent class analysis suggested better specificity of the genus-specific ITS-2 PCR compared with the more sensitive *Sm1-7*-based protocols. Accordingly, while species-specific PCR proofed to be more sensitive for *Schistosoma* DNA in blood, positive results obtained by the ITS-2-based genus-specific PCR provided a higher degree of reliability in the assessed samples from a tropical high endemicity setting. Compared to a recent assessment of *S. mansoni*-specific *Sm1-7* real-time PCR with travelers and migrants [48], both sensitivity and specificity estimations for this PCR assay in the present assessment in the Madagascan high endemicity setting suggested slightly worse diagnostic accuracy, although there is considerable overlapping of the confidence intervals (evaluation data with travelers and migrants freely accessible under [48]). This finding confirms that validation of diagnostic assays is crucial [49] and needs to be independently performed for different geographic settings.

Regarding serology, both sensitivity and specificity of IgG-based immunofluorescence and ELISA outperformed the IgM-specific assays as suggested by LCA analysis. For both immunoglobulin subclasses, the immunofluorescence assay was slightly superior as suggested by latent class analysis. Whether or not such differences justify the preference of the more laborious immunofluorescence approach over automatable ELISA in the diagnostic routine remains an individual decision. Observed poor to slight correlation of positive PCR and positive IgM results discourage the use of IgM as an activity marker of the infection in highly endemic settings like in Madagascar. The result can be explained by the facts that active infection does not necessarily mean new infection, and that reinfections may readily occur in high endemicity settings [36] like in Madagascar.

A previous assessment of the same samples for *Plasmodium* spp. [29] allowed the analysis of potential interfering effects between schistosomiasis serology and malaria serology as suggested previously [39,40,41]. Interestingly, the observed phi coefficient for concordance between malaria serology and *Schistosoma* spp.-specific serology was negligibly low and there was no big difference between the phi coefficients for serology and PCR. Accordingly, it can be concluded that the applied serological approaches may be relatively robust regarding potential interfering influence of malaria-specific antibodies.

The study has a number of limitations. First of all, its retrospective nature based on yet available residual sample materials did not allow the inclusion of diagnostic approaches other than PCR and serology from blood. Other approaches like antigen testing from urine [32], PCR from stool, urine, sperm, or bioptically acquired materials as well as traditional microscopic assessments [4] could not be added to define a more reliable composite reference standard. Secondly, the samples were more than 10 years old when the assessments were performed. Although the residual materials had been adequately stored at −80 °C prior to the assessments, effects of long-term storage on the test results cannot be completely excluded. Thirdly, the *Sm1-7*-based and the *Dra1*-based PCR approaches as well as the serological immunofluorescence assays had been developed and evaluated primarily for serum, not for EDTA plasma. Only the ELISA had been previously validated by the manufacturer for plasma. Accordingly, sample matrix-specific effects may have influenced the results as well. All those pre-analytic conditions cannot be changed but have to be considered while interpreting the study results. Further, prospective assessments with fresh sample materials are advisable. Fourthly, the combined presentation of epidemiological prevalence assessments and test evaluation results may be dissatisfying for readers just looking for the one or the other. However, test accuracy can vary at different geographic regions as discussed for the blood PCR above depending on the endemicity of target microorganisms and potentially cross-reacting non-target microorganisms [43]. In turn, test accuracy can influence study results like prevalence estimations [49], so it makes sense to evaluate diagnostic accuracy at the study site.

## 4. Materials and Methods

### 4.1. Study Design and Sample Materials

A cross-sectional study was conducted based on residual EDTA plasma samples [25,26,27,30] and nucleic acid extractions from urea-stabilized EDTA blood samples [28,29] from 1244 pregnant Madagascan women obtained between April and July 2010 in 6 different locations in Madagascar, 2 coastal and 4 in the highlands. The samples had been taken at the time of routine check-ups during pregnancy. Thereby, however, the sample acquisition was study-associated.

At the time of sample acquisition, neither routine screening nor associated treatment of schistosomiasis had been an element of routine check-ups for Madagascan pregnant women.

All residual EDTA plasma samples and nucleic acid extractions had been stored frozen at −80 °C prior to the assessments.

### 4.2. PCR Protocols

In total, three real time PCR assays were applied with the residual nucleic acid extractions from urea-pretreated blood; (i) a *Schistosoma*-ssp.-overarching PCR targeting the ITS-2 region for *Schistosoma* spp., thus detecting *S. mansoni*, *S. haematobium*, and *S. intercalatum* [31], (ii) a PCR specific for the *S. mansoni* complex targeting the highly repetitive *Sm1-7* region of *S. mansoni* [33,50]; and (iii) a PCR targeting the highly repetitive *Dra1* region of *S. haematobium* complex [34]. PCRs were run on RotorGene Q (Qiagen, Hilden, Germany) or MIC (Bio Molecular Systems, Upper Coomera, Australia) cyclers as previously described [31,33,34,48]. The *Sm1-7* assay and the *Dra1* assay were run in a one-tube duplex approach as detailed elsewhere [47]. Internal control was based upon the amplification of Phocid herpes virus DNA [51].

### 4.3. Serology

Serological assessments were performed using commercial anti-Schistosoma IgM- and IgG ELISA and immunofluorescence assays (EUROIMMUN Medizinische Labordiagnostika AG, Lübeck, Germany). While optical density values were measured for the ELISA assessments, positive slides in immunofluorescence at 1:10 dilution were additionally assessed applying the titers 1:100 and 1:1000 for quantification purposes. Previous data on *Plasmodium* spp. detections within the samples [29] were used to evaluate potential cross-reaction by calculating the phi coefficient.

### 4.4. Inclusion Or Exclusion Criteria

All available residual samples were assessed. Insufficient sample material for all assessments or lacking clinical information were no exclusion criteria.

### 4.5. Statistical Assessment

For each diagnostic method, we calculated the prevalence of positives and then compared the agreement between the tests. Association with the altitude of the assessed study sites was analyzed calculating the point biserial correlation coefficient. The proportion of concordant positives and discordant results between the serological methods and between PCR-methods and IgM-antibody detections methods were determined. Agreement (kappa) between the tests was calculated and categorized based on the category’s poor (below 0.00), slight (0.00–0.20), fair (0.21–0.40), moderate (0.41–0.60), substantial (0.61–0.80), and almost perfect (0.81–1.00) as suggested [43]. In case of concordantly positive results between genus-specific PCR and species-specific PCR, cycle threshold values were comparatively assessed. Latent class analysis (LCA) [44,52] was applied to calculate sensitivity and specificity of the serological and molecular tests. Additionally, LCA-based calculation of diagnostic accuracy-adjusted overall prevalence of schistosomiasis within the investigated population was performed. Prior to those calculations, relevant association between malaria and schistosomiasis positivity was excluded by calculating the phi-coefficient. All analyses were done applying Stata version 14 (College Station, TX, USA).

The population prevalence was estimated based on the positives within the assessed samples conducting LCA and was provided with a 95% confidence interval.

### 4.6. Ethical Clearance

Madagascan ethical clearance for the epidemiological assessment was provided by the “Comité d’Ethique de la Recherche Biomédicale auprès du Ministère de la Santé Publique de Madagascar” (No 77 MSANP/SG/-AGMED/CNPV/CERBM) as an extension of the ethical approval of the original sample collection (Autorisation 051-CE/MINSAN du 02 Novembre 2009). In addition, ethical clearance for blinded use of residual materials for test comparison and evaluation purposes was granted by the ethics committee of the Medical Association of Hamburg, Germany (registration number WF-011/19) in line with German national laws.

## 5. Conclusions

In spite of the abovementioned limitations, this study indicated a high prevalence of schistosomiasis among pregnant women in Madagascar as confirmed by blood PCR and serology [36,37] with considerable disease activity as confirmed by blood PCR [37]. Further, the study confirmed high sensitivity of *Sm1-7*-based blood PCR for *S. mansoni* complex [33]. However, less sensitive approaches like ITS-2-based PCR [31,53] may be useful for surveillance purposes in high endemicity settings as well, if optimum specificity is desired. Only slight to moderate superiority of comparatively laborious immunofluorescence-based schistosomiasis serology over automatable ELISA suggests ELISA-based surveillance as an option worth considering, in particular, if diagnostic accuracy adjusted prevalence estimation [42] is applied to calculate the true prevalence.

As a more indirect conclusion, the study showed that historic sample materials can be valuable by allowing the assessment of diagnostic assays targeting neglected tropical diseases. Insofar, the study also encourages microbiological biobanking [54,55], a strategy which has already been suggested as promising for the development of diagnostic tools for sleeping sickness [56].

## Figures and Tables

**Table 1:** Test results for serological tests searching for IgG and IgM antibodies against *Schistosoma* spp. and results of 3 different PCR approaches searching species-overarching for *Schistosoma* spp. in general, and species-specific for *S. haematobium* and *S. mansoni* in 6 different locations at Madagascar.

		Immunofluorescence Assays, % Positives *		ELISA, % Positives *		PCRs, % Positives		
Altitude	N	IgM	IgG	IgM	IgG	*Schisto-soma* spp. ^1^	*S. haematobium* ^2^	*S. mansoni* ^3^
Littoral	187	37.4%	48.7%	29.4%	37.4%	7.5%	0	32.6%
Littoral	231	12.6%	8.2%	19.9%	10.8%	0	0	24.2%
466 m	174	29.9%	30.5%	42.5%	37.4%	5.2%	0	41.4%
920 m	166	27.7%	24.7%	21.1%	38.6%	5.4%	0	36.7%
860 m	202	37.1%	51.0%	32.2%	54.0%	1.5%	0	50.0%
1280 m	194	29.4%	63.4%	29.4%	71.1%	14.4%	0	69.1%
All sites	1154	28.5%	37.3%	28.8%	40.8%	5.5%	-	42.0%
Point biserial correlationcoefficient (*p*-value)	1154	0.0672 (0.0224)	0.2360 (0.0001)	0.0270 (0.3595)	0.3330 (0.0001)	0.1183 (0.0001)		0.2614 (0.0001)

* Serological marginals in line the manufacturer’s instructions were not counted as positives. ^1^ Species-overarching PCR for *Schistosoma* spp. targeting the ITS-2 region of African *Schistosoma* spp. ^2^ *S. haematobium* PCR targeting the highly repetitive *Dra1* region of *S. haematobium* complex. ^3^ *S. mansoni* PCR targeting the highly repetitive *Sm1-7* region of *S. mansoni* complex.

**Table 2:** Comparison of test results between serological methods for the detection of schistosomiasis (immunofluorescence assay vs. ELISA, *n* = 1154).

		% Positive	% Concordant Positive	% Concordant Negative	% Discordant	Kappa (SE)	Kappa Interpretation
IF IgG	Positive	37.3%	31.2%	53.1%	15.7%	67.1% (2.9%)	Substantial
ELISA IgG		40.8%					
IF IgG	Positive and borderline	44.5%	38.0%	47.0%	15.1%	69.6% (2.9%)	Substantial
ELISA IgG		46.5%					
IF IgM	Positive	28.5%	19.8%	62.5%	17.8%	56.5% (2.9%)	Moderate
ELISA IgM		28.8%					
IF IgM	Positive and borderline	36.1%	24.4%	55.2%	20.4%	55.1% (2.9%)	Moderate
ELISA IgM		33.2%					

S.E. = standard error.

**Table 3:** Comparison of test results between serological methods for the detection of IgM antibodies and PCR methods (immunofluorescence assay vs. ELISA, *n* = 1154).

	% Positive	% Concordantly Positive	% Concordantly Negative	% Discordant	Kappa (SE)	Kappa Interpretation
IF IgM *	28.5%	19.8%	62.5%	17.8%	56.5% (2.9%)	Moderate
ELISA IgM *	28.8%					
*S. mansoni* PCR ^2^	42.3%	4.9%	57.5%	37.6%	12.3% (1.5%)	Slight
*Schistosoma* spp. PCR ^1^	5.5%					
IF IgM *	28.5%	15.9%	45.3%	38.8%	16.6% (2.8%)	Slight
*S. mansoni* PCR ^2^	42.3%					
IF IgM *	28.5%	2.5%	68.5%	28.9%	6.2% (2.0%)	Slight
*Schistosoma* spp. PCR ^1^	5.5%					
ELISA IgM *	28.8%	13.3%	42.5%	44.3%	5.0% (2.8%)	Slight
*S. mansoni* PCR ^2^	42.3%					
ELISA IgM *	28.8%	0.9%	66.6%	32.5%	−4.5% (2.0%)	Poor
*Schistosoma* spp. PCR ^1^	5.5%					

S.E. = standard error. * Borderlines were not included as positives in the analysis. ^1^ Species-overarching PCR for *Schistosoma* spp. targeting the ITS-2 region of African Schistosoma spp. ^2^ *S. mansoni* PCR targeting the highly repetitive *Sm1-7* region of *S. mansoni* complex.

**Table 4:** Test characteristics as calculated based on latent class assessment and the resulting diagnostic accuracy adjusted overall prevalence rate.

	Immunofluorescence Assays		ELISA of		PCR		
N = 1154	IgM	IgG	IgM	IgG	*Schistosoma* spp.	*S. haematobium*	*S. mansoni*
Sensitivity (0.95 CI)	0.4913 (0.4438, 0.5391)	0.8696 (0.8142, 0.9103)	0.3829 (0.3377, 0.4302)	0.8759 (0.8282, 0.9118)	0.1349 (0.1061, 0.1702)	-	0.7406 (0.6929, 0.7832)
Specificity (0.95 CI)	0.8549 (0.8221, 0.8825)	0.9647 (0.934, 0.9811)	0.7769 (0.7418, 0.8085)	0.9093 (0.8753, 0.9347)	0.9999 (0, 1)	-	0.7971 (0.7618, 0.8284)
Prevalence rate (0.95 CI)	40.4% (36.8%, 44.1%)						

**Table 5:** Phi coefficients to assess potential cross-reactions between anti-malarial and anti-*Schistosoma*-antibodies as well as comparison with malaria and schistosomiasis PCR. Data for the malaria assessments were taken from a previous publication [29].

	Immunofluorescence Assays		ELISA		PCR		
N = 1154	IgM	IgG	IgM	IgG	*Schistosoma* spp.	*S. haematobium*	*S. mansoni*
Phi coefficient *	0.0372	0.1150	0.0297	0.1417	0.1231	-	0.1006

* for concordance between malaria serology and *Schistosoma* spp. diagnostics.

## Data Availability

All relevant data are provided in the manuscript. Raw data can be made available on reasonable request.

## Appendix A. Distribution of the Excluded Samples due to Insufficient Sample Volumes on the Different Study Sides by Altitude

**Altitude**	**Total Number of Collected Samples (%)**	**Excluded Samples due to Insufficient Sample Volumes (%)**
Littoral	195 (100.0)	8 (4.1)
Littoral	251 (100.0)	20 (8.0)
466 m	197 (100.0)	23 (11.7)
860 m	203 (100.0)	1 (0.5)
920 m	198 (100.0)	32 (16.2)
1280 m	200 (100.0)	6 (3.0)
